# Unraveling the enhanced Oxygen Vacancy Formation in Complex Oxides during Annealing and Growth

**DOI:** 10.1038/srep39953

**Published:** 2017-01-16

**Authors:** Felix V. E. Hensling, Chencheng Xu, Felix Gunkel, Regina Dittmann

**Affiliations:** 1 Peter Grünberg Institut 7, Forschungszentrum Jülich GmbH, Jülich, 52428, Germany; 2 RWTH Aachen University, Institute of Electronic Materials (IWE 2), Aachen, 52056, Germany

## Abstract

The reduction of oxides during annealing and growth in low pressure processes is a widely known problem. We hence investigate the influence of mere annealing and of growth in vacuum systems to shed light on the reasons behind the reduction of perovskites. When comparing the existing literature regarding the reduction of the perovskite model material SrTiO_3_ it is conspicuous that one finds different oxygen pressures required to achieve reduction for vacuum annealing and for chemically controlled reducing atmospheres. The unraveling of this discrepancy is of high interest for low pressure physical vapor depositions of thin films heterostructures to gain further understanding of the reduction of the SrTiO_3_. For thermal annealing, our results prove the attached measurement devices (mass spectrometer/ cold cathode gauge) to be primarily responsible for the reduction of SrTiO_3_ in the deposition chamber by shifting the thermodynamic equilibrium to a more reducing atmosphere. We investigated the impact of our findings on the pulsed laser deposition growth at low pressure for LaAlO_3_/SrTiO_3_. During deposition the reduction triggered by the presence of the laser plume dominates and the impact of the measurement devices plays a minor role. During post annealing a complete reoxidization of samples is inhibited by an insufficient supply of oxygen.

The field of transition metal oxides received special attention over the last decade due to their interesting electronic, magnetic and related properties^1^,^2^. With metal oxides playing a key role in today’s research pulsed laser deposition (PLD) became one of the most common used deposition techniques. This is owed to the fact that PLD is a versatile and powerful tool for the deposition and epitaxial growth of this material class^2^,^3^,^4^.

The oxygen pressure applied during the deposition of metal oxides plays a key role for the properties of the resulting thin films^5^,^6^,^7^,^8^,^9^,^10^,^11^. For the deposition of fully oxygenated thin films, a high oxygen pressure is necessary to either compensate or inhibit the oxygen ejection from the thin film induced by the high energetic species of the plasma plume. At the same time some oxides require a low oxygen pressure during deposition in order to guarantee a low valence state of the involved cations^12^,^13^, or to obtain layer-by-layer growth, as high physical pressures result in 3D growth^10^,^14^,^15^. Thus when aiming for high quality heterostructures or superlattices the competition of crystalline perfection and stoichiometry limits the choice of materials and the achievable interface quality^10^,^16^,^17^. An example for the importance of the oxygen pressure during growth is the LaAlO_3_(LAO)/STO heterostructure. LAO/STO is well known for showing interface conductivity between two otherwise insulating materials^7^,^16^,^18^,^19^,^20^,^21^,^22^,^23^. However, if the STO substrate is reduced during low oxygen pressure deposition a strong contribution of the bulk conduction of the STO is measurable^6^,^7^, while a deposition at high oxygen pressures can result in an undesirably high resistance^18^,^20^. One of the central challenges of PLD processes is thus, the need of a low oxygen pressure to grow certain complex oxides and the accompanied undesired reduction of the thin film, the substrate or both.

We will consider this challenge closer for processes including an STO substrate, which is by far the most widely used substrate material for the growth of epitaxial oxide thin films and has the advantage of a well known defect chemistry, which is highly sensitive to the applied oxygen pressure. In the moderately low oxygen pressure regime the oxygen vacancy concentration (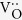) of STO is controlled by the inherent acceptor type impurities, which are omnipresent in STO, as they are impurities incorporated during manufacturing (typically Fe, Al, Mn). In this regime lowering the oxygen pressure further will result in additional oxygen vacancies, which will contribute two electrons to the conduction band resulting in an n-conductivity. This can be described by Eq. 1 in the Kröger Vink notation^24^, reflecting the oxygen pressure dependence of the electron concentration in STO^23^,^25^,^26^,^27^.


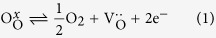


In the intermediate oxygen pressure regime the electron concentration decreases and the conductivity of STO reaches a minimum, which can be attributed to the ionic contribution of the oxygen vacancies. For high oxygen pressures the concentration of oxygen vacancies is further decreased leading to the formation of holes, eventually resulting in p-conductivity observable at elevated temperatures^23^,^25^,^26^,^27^. This process can be described by Eq. 2 in the Kröger Vink notation^24^.


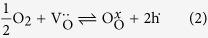


Numerous examples can be found in literature for the reduction of STO substrates during pulsed laser depositions for physical oxygen pressures below 10^−5^ mbar^6^,^7^,^11^,^16^,^17^,^18^,^20^,^21^,^22^. This opposes the results of chemically controlled reducing atmosphere annealing experiments (H_2_/Ar/O_2_ mixtures), for which the sample is, in opposite to vacuum annealing, under a physical pressure of 1 bar. These experiments determined that STO reduction, in case of inherent acceptor-type impurity content, will only take place at oxygen partial pressures below 10^−17^ mbar even at elevated temperatures^23^,^25^,^26^,^28^. The contradiction is often explained by the influence of the laser plume during growth^10^,^11^,^17^. Lee *et al*. e.g. have recently found that an increased ion bombardment *via* the laser plume results in an increased reduction^10^. Another explanation often found in literature is the oxidization of the growing thin film by the STO substrate, eventually resulting in the reduction of the substrate^29^,^30^,^31^. However, as soon as the plume is turned off one would expect the oxygen content of the sample to approach the equilibrium value and thus to reoxidize at a certain rate within the PLD chamber^21^.

The reduction of STO in PLD vacuum chambers, however, is not only observed during deposition, but also observed when annealing at physical oxygen pressures below ≈7 · 10^−7^ mbar^16^,^32^,^33^, which is in contradiction to the thermodynamic equilibrium measurements performed in chemically controlled gas mixtures or with oxygen pumps^23^,^25^,^26^. To our knowledge, no work sufficiently explained this contradiction between physical oxygen pressure and chemically controlled oxygen pressure so far. The oxygen pressures used for vacuum annealing (≈10^−6^ mbar) are considered as low pressure growing conditions. However, the oxygen pressure is comparably high considering the thermodynamical equilibrium of STO. In other words, a chemical oxygen partial pressure of 10^−6^ mbar is not reducing at all. Understanding the reduction mechanism of STO in vacuum chambers at comparably high oxygen pressures as a first step may subsequently improve the understanding of the low pressure growth of oxides by PLD. Based on this understanding it may be achievable to grow at low physical pressures, but at the same time to avoid a reduction of the substrate.

We investigate in detail the influence of some of the most common procedures typically involved in PLD processing. First we investigate the influence of contaminations which originate on the one hand from adsorbates on the holder as well as the sample and on the other hand from the gluing of samples on holders with Ag-paste. Second we investigate the influence of measurement devices attached to the PLD chamber, namely a cold cathode gauge for pressure measurement and a mass spectrometer. Being able to explain the reduction of STO during annealing, we disentangle the reduction of STO during depositions. We do so using LAO growth on STO due to its oxygen pressure sensitivity^7^,^16^,^21^,^23^. We are able to separate influences induced by the plume from influences of an insufficient supply of oxygen and influences of the measurement devices.

## Reduction of STO Substrates during Annealing

### Influence of Contaminations on the Reduction

A first hint to a possible reason for the reduction of STO at comparably high oxygen pressures are the findings of Frederikse *et al*., who observed the presence of oil of their fore pump in their vacuum chamber to accelerate the reduction process of STO^32^. Based on this observation, one may surmise that the gas mixture present when annealing in vacuum chambers may actually contain only small partial pressures of oxygen, *p*_*ox*_, resulting in a discrepancy between the measured total pressure *p* inside the chamber and the actual *p*_*ox*_, due to contaminations.

Thus, in a first step, we investigate the gas mixture present during a typical annealing process in a vacuum chamber. For this we heat STO single crystals glued on the holder using Ag-paste for 1 h in our PLD chamber with a rate of 20 °C/min to 800 °C. Utilizing the diffusion constant by de Souza *et al*.^27^ we can determine the diffusion length of oxygen vacancies to be 8 mm, when annealing at 800 °C for 1 h, and, therefore, to exceed the sample dimensions. Furthermore a surface reaction limitation can be excluded for high temperatures^34^. Therefore, we can assume that the samples are in thermodynamical equilibrium.

**Figure 1:**
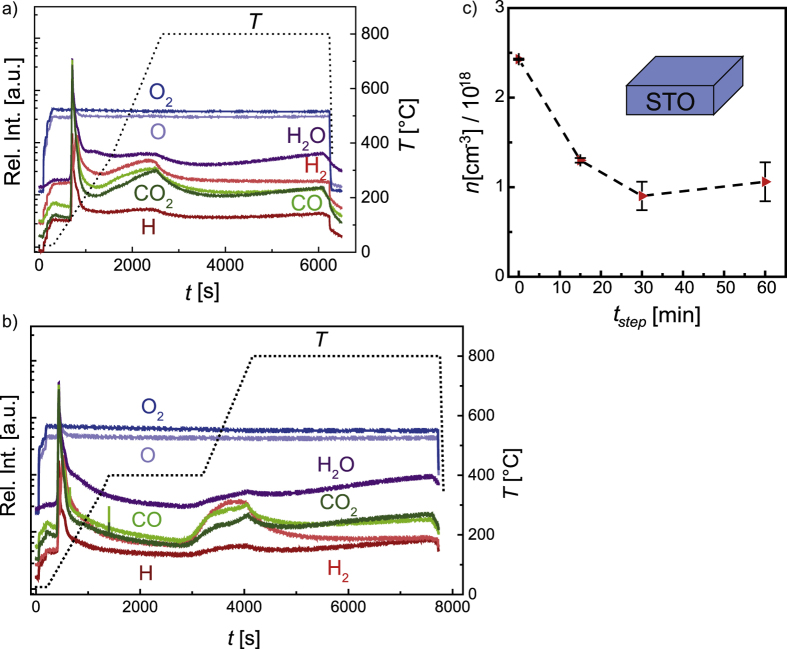
(**a**) Mass spectrometry data during thermal annealing of STO crystals inside a PLD chamber at 10^−6^ mbar without pre-annealing. The mass spectrometry graphs show the relative intensity of the measured ions (y-axis) over time (x-axis). (**b**) mass spectrometry data obtained for two-step annealing (T = 400 °C for 0.5 h pre-anneal followed by T = 800 °C for one hour). (**c**) Carrier concentration (y-axis) for different durations of the pre-annealing step (x-axis). The error bars indicate the standard deviation of the carrier concentration measured for at least two equally treated samples, verifying the reproducibility of our results. The dashed line is a guide to the eye. Mass spectrometer and cold cathode gauge were running for all acquired samples.

Some samples were treated with an additional pre-annealing step at 400 °C. We choose this temperature as it is high enough to evaporate most of the organic solvents and adsorbates, but at the same time no detectable reduction on the chosen time scales appears for STO^27^. As we will discuss, this leads to varied reduction behavior of the samples. At the end of the experiment, the samples are quenched reaching a temperature ≤350 °C within 65 s, what circumvents further changes in the samples. The background pressure before the start of the experiment is ≈3 · 10^−8^ mbar. During the experiment the pressure is set to 10^−6^ mbar with the help of a constant oxygen flow.It is measured *via* a cold cathode gauge throughout all experiments of Fig. 1. Further a mass spectrometer was running during these experiments to analyze the gas composition.

Figure 1 shows the obtained mass spectrometry data for annealing a sample 1 h at 800 °C without (a) and with (b) an additional pre-annealing step (0.5 h). In both cases starting with the appliance of the oxygen flow, the masses of O_2_ and O become the dominantly detected ones (dark blue and light blue, respectively). The simultaneous increase of the other measured species can be explained by the imperfect degree of purity of the oxygen inlet gas (purity: 99.995%). In particular we detect contaminations of H (dark red), H_2_ (light red), H_2_O (violet), CO/N_2_ (light green) and CO_2_ (dark green). Shortly after starting heating (black dotted line) a strong increase of all detected species except O and O_2_ is observed. CO and N_2_ are hard to distinguish as they have similar molecular masses. Analysis of the CO/CO_2_ ratio, however, indicates that mainly CO is present. We associate this strong increase on the one hand with the evaporation of the organic solvents of the Ag-paste, as it is less distinct for samples fixed without Ag-paste (Supplementary Figure 1), on the other hand evaporating adsorbates on the sample holder are responsible as well. The sample holder is the main source of adsorbates, as it is the only part that is transfered in and out of the chamber. The temperature of the chamber itself never exceeded 60 °C and it is thus unlikely that adsorbates situated at the chamber wall play a significant role.

After the initial heavy evaporation, the amount of contaminations decreases to a local minimum and oxygen becomes the dominating species again. When heating without an additional pre-annealing step (Fig. 1(a)) the signal slowly increases again with increasing temperature. The additional pre-annealing step at 400 °C (Fig. 1(b)) shifts the local minimum to later point in time as the amount of contaminations keeps decreasing. The additional pre-annealing step, however, cannot prevent an increase of the contaminations shortly before reaching 800 °C. After reaching 800 °C (dashed lines) there is a transient decrease of contaminations towards a nearly constant value. The amount of contaminations, especially H_2_ during the annealing step at 800 °C is lower for the case of an additional pre-annealing step. Although a significant amount of contaminations is measurable, O_2_ and O are the dominant species with and without an applied pre-annealing step.

After remaining one hour at 800 °C, the samples are quenched and a Hall measurement is carried out subsequently. The resulting carrier concentration (*n*) for samples annealed without an additional annealing step is ≈2.4 · 10^18^ cm^−3^ assuming homogeneous doping within the entire crystal (Fig. 1(c)). Remarkably, this carrier concentration corresponds to heavily reduced STO^25^. To investigate whether the contaminations are responsible for the reduction of STO we varied the duration of the additional pre-annealing step at 400 °C. The resulting *n* values for different durations of the 400 °C annealing step are depicted in Fig. 1c). A 15 minute annealing step results in a almost two times lower *n* (≈1.3 · 10^18^ cm^−3^). While still achieving a slight decrease in carrier concentration with an increase of the time towards 30 minutes (≈0.9 · 10^18^ cm^−3^) a further increase of the pre-annealing time towards 60 minutes does not change the carrier density further.

The observed behavior is surprising, because the reduction behavior of the STO single crystals indicates a local oxygen partial pressure that is well below the physical pressure of 10^−6^ mbar. This however, seems to contradict the results of the mass spectrometry experiments revealing that oxygen is the dominant component of the gas mixture. A possible explanation for O_2_ and O being the dominant species and at the same time reducing the sample is that the origin of the contaminations. The sample and its holder are the main source of contaminations, as only the sample holder is transferred from outside the chamber and the silver paste, used to fixate the sample, outgasses. However, both, mass spectrometer and oxygen inlet, are not in the direct vicinity of the sample. It is thus conceivable that contaminations react with the sample before reaching the mass spectrometer. The decrease in contaminations, achieved by the additional annealing step, results in a less reduced substrate. Thus, the contaminations play a role in the enhanced oxygen vacancy formation during vacuum annealing. We identified H and H_2_^23^,^25^,^26^ as well as CO^35^ as species responsible for this effect. The equilibria describing their oxidation behavior in the gas atmosphere are the following:


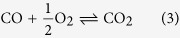



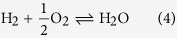


Applying those to STO under consideration of Eq. 1 results in:


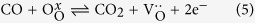



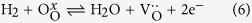


which then describes the oxidation of carbon monoxide and hydrogen in the vicinity of an oxide surface. In this case, these molecules tend to remove oxygen from the solid in order to get oxidized.

We have at this point found that the contaminations present in the PLD chamber play an important role for the reduction of STO. We were able to decrease the amount of contamination present during annealing by applying an additional annealing step resulting in a less reduced STO substrate. The pre-annealing temperature of 400 °C is high enough to enable the evaporation of most adsorbates, at the same time it is, according to literature, low enough to prevent significant release of oxygen from the substrate at the chosen time scales^27^. However STO is still reduced to a greater extend than we would expect considering chemically controlled reducing atmospheres^23^,^25^,^26^. Thus we consider other possible influences.

### Influence of Measurement Devices on the Reduction

A hint to another possible influence on the enhanced oxygen vacancy formation was recently published by Scheiderer *et al*.^36^. They found an influence of the cold cathode gauge on the reduction state of LAO/STO heterostructures in H_2_O rich atmospheres. They identified the cold cathode gauge to influence the ratio of H_2_O and its ionic derivates^36^. Thus we investigate the influence of similar measurement devices applied to our vacuum chamber, namely a cold cathode gauge and a mass spectrometer.

**Figure 2:**
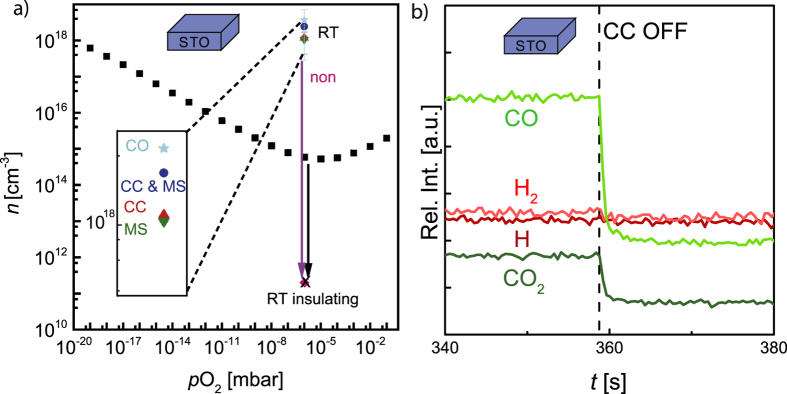
(**a**) Resulting carrier concentrations (y-axis) for annealing one hour at 10^−6^ mbar and 800 °C with different measurement devices running. CC meaning cold cathode gauge and MS mass spectrometer, CO representing a CO/O_2_ atmosphere. The error bars indicate the standard deviation of the carrier concentration measured for at least two equally treated samples, verifying the reproducibility of our results. The carrier concentration is plotted into a chemically controlled thermodynamic equilibrium graph with the *p*O_2_ as x-axis for better comparability^23^. The inset shows details of the resulting sheet carrier concentration for different running measurement devices without the error bars. The pink arrow indicates the change for switched off measurement devices, the black arrow indicates the change for measuring at room temperature^37^. The crossed out data point indicates a sheet carrier concentration below the measurement limit. (**b**) Relative intensity of selected ions (y-axis) over the time (x-axis) measured via mass spectrometry in the moment of the cold cathode gauge turn off (dashed line) measured at the same parameters. Dark red representing H, light red H_2_, light green CO and dark green CO_2_.

Figure 2(a) depicts the carrier concentration of different STO single crystals annealed for one hour at 800 °C and 10^−6^ mbar without pre-annealing step in comparison to chemically controlled thermodynamic equilibrium data from our earlier work (black squares)^23^. The thermodynamic equilibrium data is acquired in chemically controlled reducing atmospheres. As the pressure for these experiments is constantly 1 bar neither a cold cathode gauge nor a mass spectrometer are used. In order to draw this comparison, our equilibrium conductivity data^23^ is converted into the corresponding carrier concentration utilizing the mobility formula provided by Moos *et al*.^25^. These data serve as reference for the carrier density expected for STO in chemically controlled thermodynamic equilibrium. The data points represent the carrier concentration of the sample with a running cold cathode gauge and mass spectrometer (blue dot), a running cold cathode gauge (red triangle), a running mass spectrometer (green triangle), and without any of both devices running (crossed pink square).

The highest sheet carrier concentration is achieved when both measurement devices are running (≈3 · 10^18^ cm^−3^). With only one measurement device running *n* is considerably smaller (≈1 · 10^18^ cm^−3^), but, considering the equilibrium data, still in the magnitude of highly reduced STO. Remarkably, when no measurement device is running during annealing, *n* is lower than the detection limit of the Hall probe (10^11^ cm^−3^). The fact that the carrier concentration is even lower than the equilibrium data is easily explainable considering the measurement methods. The equilibrium conductivity data is measured at elevated temperatures, while our measurements are performed at room temperature. Considering the temperature dependence of small carrier concentrations, the corresponding data is below the threshold for n-type conduction in the quenched state (about 10^15^ cm^−3^)^37^. Therefore, we can conclude that the sample is not reduced.

These results are surprising as they prove the attached measurement devices as being responsible for the reducing atmosphere in the vacuum chamber. A possible reason can be extracted from Fig. 2(b). It shows again the mass spectrometry data for CO_2_ (dark green), CO (light green), H_2_ (light red) and H (dark red) obtained at 10^−6^ mbar. We choose these species as we identified them as responsible for the reduction of STO (Eqs 5 and 6). When the cold cathode gauge is turned off (dashed line), the signals of CO_2_ and CO decrease. Noticeably, the amount of CO decreases to a stronger extend than the amount of CO_2_, while there is no change observable for H_2_ and H. Thus the cold cathode gauge shifts the CO/CO_2_ equilibrium towards the reducing CO (Eq. 5). This differs from the findings of Scheiderer *et al*. as they found a shift in the ratio of H_2_O and its derivates. However, in contrast to our experiments they worked in H_2_O rich atmospheres, which explains the different observations^36^.

To verify CO as the contamination mainly responsible for the reduction we annealed samples in a CO/O_2_ mixture (turquois star). A constant flux of CO is applied matching the amount of CO originating from the cold cathode gauge identified in Fig. 2(b). During the annealing the mass spectrometer and the cold cathode are switched off. Samples annealed this way show a carrier concentration in the same regime as samples with both the measuring devices switched on (≈3 · 10^18^ cm^−3^). The surplus of CO originating from the cold cathode gauge is thus likely to be responsible for the reduction of STO at comparably high oxygen pressures.

Both measurement devices work based on ionization. This working principle is likely to be responsible for the shift of the CO/CO_2_ ratio towards CO. According to Eq. 3 this results in a lowered effective oxygen partial pressure 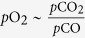. As a result of Eq. 5 one then expects a stronger reduction of the STO crystal when exposed to this atmosphere. Additionally it is conceivable that the ionization can influence the kinetics of the redox-reaction at the sample surface in a way that the removal of oxygen from the sample gets favorable^38^. In conclusion the cold cathode and mass spectrometer are primarily responsible for the unexpected reduction of STO at relatively high oxygen pressures; probably due to their working principle, which is based on ionization. Their influence outweighs the influence of the contaminations found in Fig. 1, as it is possible to avoid reduction even without an additional annealing step. This is perspicuous considering the contaminations change the carrier concentration by a factor of three, while the measurement devices eventually change the carrier concentration by at least 6 orders of magnitude.

## Relevance for low Pressure Depositions

### Role of the Plasma Plume

After having addressed the reduction behavior of STO upon vacuum annealing, we discuss possible implications for actual PLD growth processes. For this, we choose LAO grown on STO as it is a very *p*_*ox*_ sensitive system. This system shows interface conduction, when the STO substrate is not reduced, and additional bulk conduction, when STO is reduced^7^,^16^,^21^,^23^. Thus LAO/STO has the advantage of a measurable sheet carrier concentration at room temperature even if the STO substrate is not reduced. The interface conductivity is usually described by a sheet carrier concentration *n*_*S*_, thus this quantity is further used instead of *n*. A *n*_*S*_ of 1 · 10^14^ cm^−2^ is usually expected for interface conduction^18^,^19^,^21^, while a *n*_*S*_ clearly above this value corresponds to contribution from the bulk conduction of the reduced STO substrate^6^,^7^.

**Figure 3:**
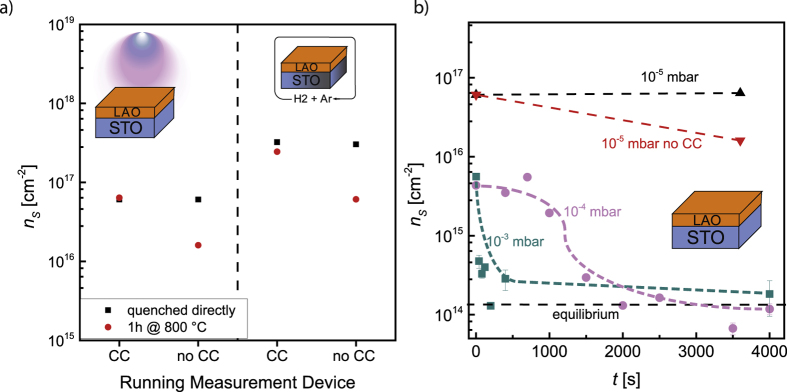
(**a**) Sheet carrier concentration of LAO/STO heterostructures for the case of a running cold cathode gauge (CC) and a turned off one, respectively. The left panel shows the sheet carrier concentration (*n*_*S*_) of LAO/STO grown at 10^−5^ mbar, when quenched directly after deposition (black) and subsequently annealed for one hour (red). The right panel shows *n*_*S*_ of LAO/STO samples, which were *ex situ* reduced in equilibrium experiments (black) and subsequently annealed in the PLD chamber under the same conditions as the grown ones (red). (**b**) depicts the annealing experiments at 10^−3^ mbar (turquois) and 10^−4^ mbar (pink), respectively. The results of our annealing experiments at 10^−5^ mbar with cold cathode (CC) (black) and without cold cathode (red) are included for comparison. The dashed black line marks the expected sheet carrier concentration for 2D interface conduction^6^,^7^.

To unravel whether the reduction of the STO substrate during LAO growth is triggered by the measurement devices, as it is for mere thermal annealing, or by the deposition itself^10^,^11^,^17^,^29^,^30^ we deposited 8 monolayers LAO on STO with a laser fluence of 1.3 J · cm^−2^ and a frequency of 1 Hz at 10^−5^ mbar and 800 °C with and without an applied cold cathode gauge. The mass spectrometer was not applied in any case. The black squares in the left panel of Fig. 3(a) represent the resulting sheet carrier concentration of the LAO/STO heterostructure quenched after deposition with and without applied cold cathode gauge. For both cases the sheet carrier concentration is ≈6.1 · 10^16^ cm^−2^ and thus in the regime of bulk contribution.

As the application of the cold cathode gauge does not influence the resulting sheet carrier concentration for samples quenched directly after deposition its influence during depositions is neglectable. The influence of the plasma plume and the oxidization of the grown film *via* the substrate, respectively, dominate the reduction of the STO substrate.

### Reoxidization during Post-Annealing

In our earlier work we have shown that it is possible to reoxidize the STO substrate reduced during growth by annealing after deposition for a sufficient time at growth conditions for pressures ≥10^−4^ mbar (Fig. 3(b))^21^. Until now this was not possible for lower pressures. As we have shown that the cold cathode gauge has a crucial influence on the reduction state of STO for thermal annealing, we investigate its influence during the post-annealing process and attempt to reoxidize the STO substrate at lower pressures.

The red squares in the left panel of Fig. 3(a) represent the resulting sheet carrier concentration when annealing for 1 h at deposition conditions with and without applied cold cathode gauge. The annealing time of 1 h ensures, as before, a diffusion length exceeding the sample dimensions^27^. The temperature of 800 °C further allows the exclusion of a surface reaction limitation^34^. While there is no change of the sheet carrier concentration, when the cold cathode gauge is applied (≈6.1 · 10^16^ cm^−2^), post-annealing without an applied cold cathode gauge results in a decrease of the sheet carrier concentration (≈1.6 · 10^16^ cm^−2^). This value, however, still corresponds to a contribution from the bulk conduction of the reduced STO substrate^6^,^7^.

To elucidate the extend of reoxidization the sheet carrier concentrations are compared to our prior annealing experiments in Fig. 3(b)^21^. Although reoxidization takes place to certain extend at 10^−5^ mbar, when the cold cathode gauge is not applied, the STO substrate is far from complete reoxidization. Working with pressures *p* ≥ 10^−4^ mbar, however, eventually results in a complete reoxidization^21^. Our explanation for this discrepancy is that for *p* ≤ 10^−5^ mbar reoxidization is hindered by a lack of oxygen species reaching the sample surface. In opposite to oxidation in chemically controlled atmospheres, where oxygen is constantly delivered *via* an equilibrium, the oxygen consumption of the reoxidization of the STO in vacuum chambers is not compensated adequately.

### Oxidization of *ex-situ* reduced Samples

To verify that oxidization is hindered by a lack of oxygen species reaching the sample surface we annealed *ex-situ* reduced samples. This allows us to exclude lasting influences of the deposition on the substrate like induced cation defects or the creation of a chemically broad interface^10^,^39^,^40^.

For this purpose we grew LAO thin films on STO at oxidizing conditions (*p* = 10^−2^ mbar) to avoid a reduction of the STO substrate^16^,^21^. Subsequently we reduced the samples in *ex-situ* equilibration experiments for 90 minutes at 1000 °C and *p*_*Ox*_ ≤ 10^−20^ mbar. The *p*_*Ox*_ for *ex-situ* reduction is adjusted by a 4% H_2_/Ar mixture resulting in a shift of the equilibrium described by Eqs. 4 and 6. This shift, eventually, result in a highly reducing atmosphere.

The sheet carrier concentration resulting from the *ex-situ* reduction is represented by the black squares in the right panel of Fig. 3(a) (3.1 · 10^17^ cm^−2^). The *ex-situ* reduced samples are subsequently annealed inside the PLD chamber for 1 h at 10^−5^ mbar and 800 °C with and without applied cold cathode gauge. These parameters ensure comparability with the prior experiments. The resulting sheet carrier concentration is represented by the red dots in the right panel of Fig. 3(a). While, again, there is nearly no change for the sample with an applied cold cathode gauge (≈2.4 · 10^17^ cm^−2^), reoxidization is observed for the sample annealed without an applied cold cathode gauge (≈0.6 · 10^17^ cm^−2^).

The sheet carrier concentrations of the *ex-situ* (≈6.1 · 10^16^ cm^−2^) and *in-situ* (≈1.6 · 10^16^ cm^−2^) reduced samples, which were subsequently annealed for 1 h without an applied cold cathode gauge, differ significantly. This proves that the reached state is not the result of lasting deposition effects, but the result of a lack of oxygen species reaching the sample surface during post-annealing and thus hindering a complete reoxidization. The experiments in this section also underline the severe influence of the cold cathode on the reduction state of the STO substrate, as annealing for 1 h with an applied cold cathode gauge leads to no reoxidization at all.

## Conclusion

To summarize, we were able to unravel the discrepancy of the oxygen pressure required to achieve reduction of STO single crystals found in literature, when comparing vacuum annealing and annealing in chemically controlled reducing atmospheres. We ascribe a minor role to the residual gases present in vacuum processes, as we are able to decrease the level of reduction by decreasing the amount of contaminations. More important, the reduction of STO mainly originates from the attached measurement devices, namely a cold cathode pressure gauge and a mass spectrometer. Both devices are based on ionization. The ionized species on the one hand shift the CO/CO_2_ equilibrium to the reducing CO and on the other hand may accelerate the surface redox reaction. These effects in combination enable the reduction of STO at pressures as high as 10^−6^ mbar. As soon as the devices are not attached no reduction takes place.

However, during depositions the attached measurement devices lose in importance as the reduction triggered by the deposition dominates the reduction state of the STO substrates. During post-annealing at pressures ≤10^−5^ mbar samples are only reoxidized, if no measurement device is attached. The STO substrate is, however, not completely reoxidized, even without an attached cold cathode gauge due to a lack of oxygen species reaching the sample surface and thus hindering a complete reoxidization.

We provided further understanding of oxidization/reduction during PLD processes at low oxygen pressures and eliminated the contradiction concerning the reduction of STO substrates during deposition and its well known defect equilibrium. Our recommendation is to consider the role of the cold cathode, when post-annealing samples during PLD processes. Moreover it should also be considered for other thermal annealing processes carried out in vacuum chambers.

## Methods

STO single crystals by *Crystec* were TiO_2_ terminated and subsequently annealed at 950 °C for 2 h in air. They were glued on an *Omicron* holder using Ag-paste by *Plano*. An IR-Diode laser heater with a wavelength of 925 nm was used inside the PLD chamber. The used cold cathode gauge for pressure measurement was a *Pfeiffer Vacuum Compact Cold Cathode Gauge* and the mass spectrometer a *Pfeiffer Vacuum PrismaPlus*. Hall measurement were carried out with a *Lakeshore 8400 Series*.

## Additional Information

**How to cite this article:** Hensling, F. V. E. *et al*. Unraveling the enhanced Oxygen Vacancy Formation in Complex Oxides during Annealing and Growth. *Sci. Rep.* **7**, 39953; doi: 10.1038/srep39953 (2017).

**Publisher's note:** Springer Nature remains neutral with regard to jurisdictional claims in published maps and institutional affiliations.

## Supplementary Material

Supplementary Figure 1
