# Supplementary material for: Unraveling the enhanced Oxygen Vacancy Formation in Complex Oxides during Annealing and Growth
Source: Sci Rep. 2017 Jan 16;7:39953. doi: 10.1038/srep39953 (PMC5238382; doi:10.1038/srep39953)
Supplement: Supplementary Figure 1 [file srep39953-s1.pdf]

Unraveling the enhanced Oxygen Vacancy Formation in Complex Oxides during Annealing and Growth

Felix V. E. Hensling[1,*], Chencheng Xu[1], Felix Gunkel[2], and Regina Dittmann[1]

[1]Peter Grünberg Institut 7, Forschungszentrum Jülich GmbH, Jülich, 52428, Germany
[2]RWTH Aachen University, Institute of Electronic Materials (IWE 2), Aachen, 52056, Germany
[*]f.hensling@fz-juelich.de

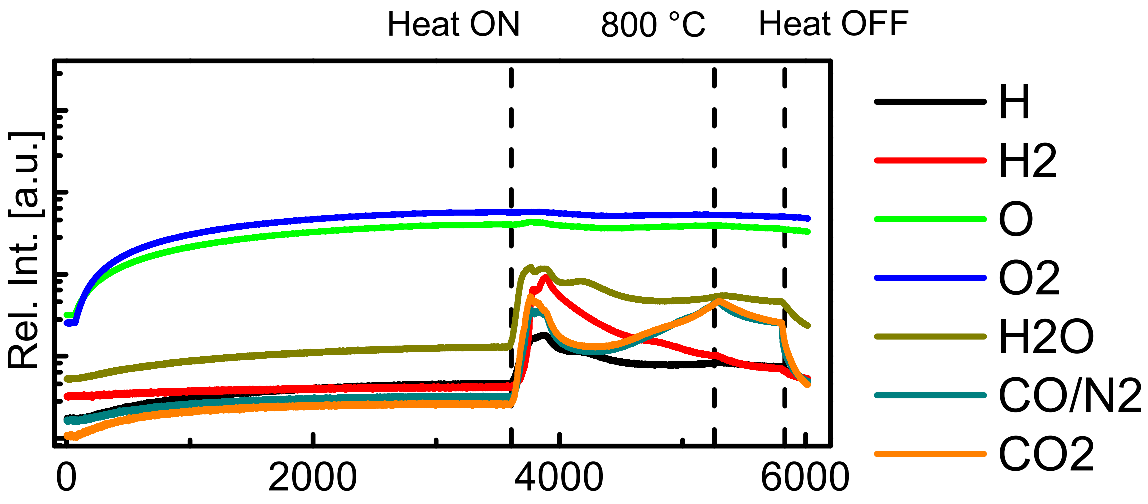

Supplementary Figure 1. Mass spectrometry data for a holder without the usage of silver paste. The y-axis represents the relative intensity of the species, the x-axis the time in seconds. In comparison to the mass spectrometry data shown in the paper the amount of residual gases is lower. Samples annealed with this alternate holder design will nonetheless be reduced.
